# Effects of Visual Training of Approximate Number Sense on Auditory Number Sense and School Math Ability

**DOI:** 10.3389/fpsyg.2020.02085

**Published:** 2020-08-27

**Authors:** Melissa E. Libertus, Darko Odic, Lisa Feigenson, Justin Halberda

**Affiliations:** ^1^ Department of Psychology and Learning Research and Development Center, University of Pittsburgh, Pittsburgh, PA, United States; ^2^ Department of Psychological and Brain Sciences, Johns Hopkins University, Baltimore, MD, United States; ^3^ Department of Psychology, The University of British Columbia, Vancouver, BC, Canada

**Keywords:** approximate number system, training, math ability, modality-independent, individual differences

## Abstract

Research with children and adults suggests that people’s math performance is predicted by individual differences in an evolutionarily ancient ability to estimate and compare numerical quantities without counting (the approximate number system or ANS). However, previous work has almost exclusively used visual stimuli to measure ANS precision, leaving open the possibility that the observed link might be driven by aspects of visuospatial competence, rather than the amodal ANS. We addressed this possibility in an ANS training study. Sixty-eight 6-year-old children participated in a 5-week study that either trained their visual ANS ability or their phonological awareness (an active control group). Immediately before and after training, we assessed children’s visual and auditory ANS precision, as well as their symbolic math ability and phonological awareness. We found that, prior to training, children’s precision in a visual ANS task related to their math performance – replicating recent studies. Importantly, precision in an auditory ANS task also related to math performance. Furthermore, we found that children who completed visual ANS training showed greater improvements in auditory ANS precision than children who completed phonological awareness training. Finally, children in the ANS training group showed significant improvements in math ability but not phonological awareness. These results suggest that the link between ANS precision and school math ability goes beyond visuospatial abilities and that the modality-independent ANS is causally linked to math ability in early childhood.

## Introduction

Educated adults and children use at least two systems to represent and process numerical information: an approximate number system (ANS), which allows observers to form imprecise estimates of the number of items in a collection without verbally counting (Dehaene et al., 1998), and an exact number system, which allows them to represent precise cardinalities and which is essential for formal mathematics (Dehaene, 1992). As educated adults, we use both of these systems daily. For example, we might use our ANS to quickly estimate whether we have fewer than 10 items in our shopping cart, whereas we might use our exact number system to make sure we receive the correct change after paying for our groceries.

Although both the ANS and exact number representations support numerical reasoning, they represent number differently. The ANS outputs a noisy sense of numerosity and, as a result, the ease with which we can discriminate one number from another using the ANS depends on the ratio between the quantities. For example, observers who are briefly shown 5 blue and 10 yellow dots (i.e., a ratio of 2 when dividing the larger by the smaller number) have little difficulty in deciding that there are more yellow than blue dots. However, if shown five blue and six yellow dots (i.e., a ratio of 1.2), many observers struggle to identify the more numerous quantities without serially counting. The fact that close quantities are more difficult to discriminate shows that ANS representations are imprecise. The degree of this imprecision varies both across development (Lipton and Spelke, 2003; Halberda and Feigenson, 2008; Halberda et al., 2012; Odic, 2018) and across individuals (e.g., Halberda et al., 2008; Libertus and Brannon, 2010). Exact number representations, on the other hand, are precise and allow for exact comparisons (e.g., allowing observers to represent that six is exactly one more than five; and 106 is exactly one more than 105). Furthermore, whereas the ANS is present from birth (Izard et al., 2009) and is found in non-human animals, including fish (Dadda et al., 2009), rodents (Meck et al., 1985), and primates (Cantlon and Brannon, 2006), the exact number system is uniquely human and is acquired slowly over the course of development as children learn to count (Wynn, 1992; Feigenson et al., 2004; Carey, 2009).

Despite their phylogenetic and ontogenetic differences, recent work suggests that primitive numerical approximation abilities and symbolic, school-based mathematical abilities may be linked (see Chen and Li, 2014; Fazio et al., 2014; Schneider et al., 2017, for meta-analyses). For example, Halberda et al. (2008) found that individual differences in adolescents’ ANS precision (i.e., their ability to rapidly decide which of two dot arrays was more numerous) related to their school math abilities tested even back in kindergarten. Subsequent work found that this relation is present prior to formal math instruction (Libertus et al., 2011; Bonny and Lourenco, 2013) and is maintained throughout adulthood (Lyons and Beilock, 2011; DeWind and Brannon, 2012; Halberda et al., 2012; Libertus et al., 2012; Lourenco et al., 2012). ANS precision also predicts future math ability. For example, ANS precision measured at 6 months of age (or measured in the early preschool years) predicts later symbolic math performance (for a review, see Mazzocco et al., 2011; Feigenson et al., 2013; Starr et al., 2013; Libertus et al., 2013a). And finally, experimental studies suggest that training of the ANS can improve subsequent symbolic math performance in adults and children (Park and Brannon, 2013, 2014; Hyde et al., 2014; Park et al., 2016; Wang et al., 2016; but see Lindskog and Winman, 2016; Merkley et al., 2017; Szucs and Myers, 2017, for critiques of these training studies).

Given that approximate numerical intuitions are shared across a wide range of animal species whereas exact number concepts are uniquely human, findings suggesting a link between the evolutionarily ancient ANS and modern school mathematics abilities have been surprising, and the nature of this link has been vigorously debated. Whereas some believe that the ANS plays an important causal role in symbolic math learning (Dehaene, 1997; Starr et al., 2013; Park and Brannon, 2014), others suggest that the relation between the ANS and math performance does not implicate primitive numerical representations in supporting symbolic math. There are at least two versions of this view. One is that tasks designed to measure numerical approximation abilities instead measure the ability to represent one or more non-numerical dimensions of continuous extent in visual arrays, like density, cumulative surface area, or convex hull (Ginsburg and Nicholls, 1988; Vos et al., 1988; Allik and Tuulmets, 1991; Durgin, 1995; Tokita and Ishiguchi, 2010b; Dakin et al., 2011; Tibber et al., 2012; Gebuis et al., 2016; Leibovich et al., 2016). Indeed, visual aspects of stimulus arrays have been shown to affect ANS performance. For example, numerical discrimination is often better when the more numerous array is larger in total surface area or is denser (Rousselle et al., 2004; Halberda and Feigenson, 2008; Rousselle and Noel, 2008; Soltesz et al., 2010; Fuhs and McNeil, 2013). These findings have led some to suggest that “the existence of an approximate number system that can extract number independent of the visual cues appears unlikely” (Gebuis and Reynvoet, 2012, p. 642; for further discussion of this view, see Odic and Starr, 2018; Halberda, 2019). According to this view, the correlation between ANS tasks and symbolic math performance – and the improvement in symbolic math seen after ANS training – reflects a link between the processing of continuous magnitude dimensions (or the executive functions required to use such cues; see Gilmore et al., 2013) and math ability, rather than between approximate number and math ability.

A related claim is that general visuospatial processing skills mediate the relation between approximate and exact math. Even if observers engage numerical representations in visual ANS tasks, they must also engage in spatial attention, visual-spatial segregation, and executive functioning. For example, Cheyette and Piantadosi (2019) recently showed that visual fixation patterns predicted adults’ non-symbolic number estimates (see also Paul et al., 2017). Some have suggested that it is variation in such aspects of visual processing that is measured by ANS tasks and that this in turn predicts math performance, rather than variation in an abstract, nonverbal “number sense.” Several lines of work suggest that visuospatial abilities may be involved in both numerical approximation and symbolic math. First, visuospatial abilities – and especially spatial attention – also are important for symbolic math. Children who are better at sustaining attention in a visual object tracking task show better symbolic math performance (Anobile et al., 2013), as do children with better visual working memory (Bull et al., 2008; LeFevre et al., 2010) and children with stronger executive function skills (Bull and Scerif, 2001; Mazzocco and Kover, 2007; Bull et al., 2008). Second, there are well-known neural links between visuospatial abilities and numerical processing, with both visuospatial tasks and numerical tasks frequently activating the intraparietal sulcus (IPS; Hubbard et al., 2005; Kaufmann et al., 2008; Zago et al., 2008; Dehaene, 2009). Activity in the IPS during a visual working memory task has been shown to predict symbolic math performance 2 years later (Dumontheil and Klingberg, 2012). Third, mathematical learning disability (i.e., developmental dyscalculia) has been linked to deficits in visuospatial processing (Rosenberg, 1989; von Aster and Shalev, 2007) and developmental impairment due to Williams syndrome or extremely premature birth is also associated with simultaneous visuospatial and numerical deficits (Molko et al., 2003; Hellgren et al., 2013; Libertus et al., 2014, 2017). From these findings, some have concluded that ANS tasks and symbolic math tasks both draw on visuospatial processing, but have no deeper link than that (Tibber et al., 2013; Zhou et al., 2015).

To date, this debate about the role of visuospatial abilities in the ANS-math link remains unresolved. At its center is a fundamental methodological challenge: it is impossible to dissociate all visuospatial abilities within the context of a visual, non-symbolic number comparison task. Because visual features are the means by which visual number information is presented, there will always be some visual confound that could explain the link between performance in a visual ANS task and performance in a math task. But while no visual ANS task will ever be free of all confounds with all possible visual dimensions, there is an alternative and complementary methodological solution: measuring ANS precision in a non-visual task, to eliminate visual processing altogether. Previous research suggests that the ANS readily uses both visual and auditory information as input. Izard et al. (2009) showed that newborn infants match the approximate number of tones they hear with the approximate number of objects they see. Six-month-old infants can discriminate visual arrays of objects that differ by a ratio of 2:1, such as 8 and 16 dots (Xu and Spelke, 2000), and sequences of tones that differ by the same ratio (Lipton and Spelke, 2003), whereas they fail at discriminating a ratio of 1.5 in both modalities. The same ratio-dependent performance is observed when 6-month-olds use sequences of tones to predict how many visual objects will appear (Feigenson, 2011). And, adaptation to visual number stimuli extends to auditory stimuli and vice versa (Arrighi et al., 2014). In addition, the ANS supports approximate arithmetic across sensory modalities: Barth et al. (2005, 2006, 2008) demonstrated that children and adults can add and subtract sequences of sounds and visually presented stimuli (e.g., adding approximately six sounds to approximately 12 dots), without any performance cost compared to when computing within a single modality. Visual and auditory numerical approximations also appear to activate the same fronto-parietal brain networks in adults (Piazza et al., 2006), and the same groups of neurons have been found to encode auditory and visual numerosity in monkeys (Nieder, 2012). And, in the total absence of visual experience, completely normal ANS functioning remains intact in audition in congenitally blind individuals who have never processed number visually (Kanjlia et al., 2018).

These findings demonstrate that infants, children, and adults represent and process approximate number from visual or auditory input, suggesting that the ANS is abstract and modality-neutral. Of course, numerical information presented in any sensory modality will always be confounded with some aspect of the stimulus. In auditory sequences, numerosity may be confounded with total duration, presentation rate, or total auditory energy. However, it is important to note that auditory and visual confounding variables are independent. When an array of dots is presented visually and simultaneously, average dot size, total surface area, total perimeter, visual density, and convex hull are salient perceptual dimensions that may be confounded with number. In contrast, in a serial auditory presentation of tones, average tone duration, total sequence duration, and tone rate may be confounded with number. These confounds are perceptually distinct, not only in terms of the sensory system in which they are perceived but also in whether they require integrating over spatial vs. temporal information. As a reflection of this distinction, individual differences in visual processing tasks often dissociate from those in auditory/verbal processing tasks (Shah and Miyake, 1996), and visual and auditory attention appear to be subserved by separable brain regions (Bushara et al., 1999). Because the confounding dimensions for vision and audition are largely independent, an observed correlation between visual and auditory ANS precision would point to a shared resource above the level of modality-specific processing.

In the current study, we had three aims. The first was simply to ask whether individual differences in the numerical approximation of visual stimuli correlate with individual differences in the numerical approximation of auditory stimuli. The second aim was to ask whether training visual approximate number discrimination would enhance not only visual ANS precision but also ANS precision in an untrained auditory number task (i.e., cross-modal transfer). The third aim was to ask whether the link between ANS precision and school math abilities reflects individual differences in an abstract, modality-neutral sense of approximate number, or if instead the correlation is explained entirely by a link between math and visuospatial abilities (i.e., subsymbolic-symbolic transfer). Kanjlia et al. (2018) found that ANS precision for auditory sequences correlated with math performance in blind and sighted adults. But, it remains unknown whether there is a visually independent link between the ANS and math ability early in development. To answer these questions, we trained 6-year-old children for 5 weeks on either a visual ANS task or a control phonological awareness task. We measured children’s visual and auditory ANS precision, as well as performance on standardized math and phonological awareness tests, immediately before and after the training. If the ANS is amodal and causally related to math abilities, we expect to observe four findings. First, visual and auditory ANS precision should correlate prior to training. Second, visual ANS training but not phonological training should improve both visual and auditory ANS precision. Third, auditory ANS precision should – like visual ANS precision – predict math performance. And, fourth, visual ANS training but not phonological training should improve subsequent math abilities.

## Materials and Methods

We examined the relation between visual numerical approximation, auditory numerical approximation, and symbolic math performance in 6-year-old children. We focused on this age because we were interested in the role of ANS representations in children who are just beginning formal instruction in mathematics. Before the training, all children completed a visual approximate number discrimination task, an auditory approximate number discrimination task, a standardized math task, and a standardized phonological awareness task.

Next, children were randomly assigned to one of two training groups: visual ANS training or phonological awareness training. We used a visual approximate number comparison task as our number training task because it has been shown to correlate with school math performance (see Chen and Li, 2014; Fazio et al., 2014; Schneider et al., 2017, for meta-analyses). The phonological awareness training group served as our active control, so that we could assess how ANS precision and math ability changed simply as a function of time and/or as a function of completing several weeks of a computer-based training game. We chose phonological awareness because it requires children to carefully process sequential auditory information (a skill also required by our auditory ANS task), because the training tasks were structured as *n*-alternative forced choice (which was the same structure required by the auditory and visual ANS tasks) and because phonological awareness has been shown to be a significant predictor of later reading and spelling skills (Lundberg et al., 1980).

All children were given a pre-programmed laptop computer to use at home, and parents were instructed on how to help administer the assigned training program roughly three times per week for 5 weeks (see below). Following this 5-week training, children were again tested on the visual approximate number discrimination task, the auditory approximate number discrimination task, the standardized math task, and the standardized phonological awareness task.

Previous research has found links between math ability and nonverbal intelligence, as well as between math ability and inhibitory control (Blair and Razza, 2007; Kyttala and Lehto, 2008), and some have claimed that individual differences in approximate number tasks reflect individual differences in inhibitory control (Gilmore et al., 2013). We, therefore, assessed these general cognitive abilities to ask whether any of our effects could be explained by individual differences in IQ or inhibitory control.

### Participants

Eighty-five children who were previously recruited as part of a larger, longitudinal study on children’s mathematics and language development took part in this study (see Libertus et al., 2011, 2013a,b). Data from 10 of these children had to be excluded because the children were inattentive during a majority of at least one of the testing sessions (i.e., they repeatedly showed signs of inattention such as answering questions without looking at the test materials, answering questions before hearing the complete prompt, or requiring many repetitions of a prompt). In addition, three children completed pre-training testing and some training but were unavailable for the post-training testing, and four children completed pre-training and post-training testing but completed fewer than 10 training sessions. Thus, 68 children (mean age = 6 years, 2 months, *SD* = 243 days; range: 4 years, 11 months – 7 years, 11 months; 34 girls) contributed data to the final analyses reported here. Thirty-three children completed the visual ANS training (mean age = 6 years, 3 months) and 35 children completed the phonological awareness training (mean age = 6 years, 1 month).

Most of the children came from families of middle to high socio-economic status. Parents of all children provided written informed consent prior to their child’s participation as approved by the Institutional Review Board at Johns Hopkins University, and children provided verbal assent before each testing session. All children received a small gift (e.g., small toy or book) to thank them for their participation after each testing session.

### Materials and Procedure

#### Training Procedure

Children were randomly assigned to either the visual ANS or the phonological awareness (active control) training group. After participating in a series of pre-training tests (described below), each child received a 15-in Asus X53U laptop preloaded with the assigned training game. A trained experimenter went to children’s homes and instructed parents and children on how to play the assigned game. Parents were told that children were to complete one session of the ANS training every other day (comprised of three training blocks, lasting a total of approximately 15 min per training day) or one level of each of the three phonological awareness training games (also lasting a total of approximately 15 min per training day) for 5 consecutive weeks, for a total of 16 sessions. Parents were told that if their child missed a session, they were to play 2 days in a row. Parents were instructed to help their child start the computer and the game if necessary, but to avoid helping them solve the task in any way. To monitor compliance with this training protocol, the game software saved the date and time of each session; additionally, parents were given a paper chart to be filled out each time their child completed a training session. An experimenter telephoned parents about once a week to monitor training completion and to address any questions or concerns.

##### Approximate Number System Training

Each ANS training session consisted of three 5-min blocks of a visual non-symbolic number comparison task, followed by a short cartoon movie (≈3 min) that was included as a reward to increase children’s motivation to complete each training session. To maintain children’s interest, each block contained a different set of visual stimuli (see details below), and between blocks, a screen informed children about their progress through the session.

On each trial, children saw two arrays of items presented side by side and had to decide which array contained more items. The number of items in each array ranged from 5 to 21. For each block, five test trials were drawn from within each of seven numerical ratio bins: 1.11, 1.14, 1.17, 1.25, 1.5, 2, and 3 (where a ratio of 1 was equality and a ratio of 1.5 might be, for example, 8 vs. 12 items) – hence, children completed a total of 105 numerical discriminations in each of the 16 training sessions (i.e., 35 trials per block for three blocks per session), or about 1,680 trials in all. Unlike in the visual ANS precision task that children completed during pre‐ and post-training (see below), the ANS training blocks contained arrays of differently shaped and colored objects, as well as clipart images of animals and toys. This was done to maintain children’s interest in the task, and also to help children recognize that the ordinal relation between the arrays was independent of features of the arrays like color and item shape. To encourage children to focus on approximate number and ignore the non-numerical perceptual features of the arrays, the density and the cumulative surface area of the items were correlated with number in one-third of the blocks (i.e., the more numerous array was denser and had a larger cumulative surface area across items), anti-correlated in another third, and equated between the two arrays for another third. This aspect of block type was pseudo-randomized before training, but the order was identical for all children. Thus, throughout training, visual cues to number were manipulated, but not entirely controlled – children could potentially use visual density, convex hull, area, or any other visual cue to solve the task, depending on the visual controls for the particular block of trials they were in. However, if children’s performance during training was based on a purely visual cue and not on a domain-general sense of number, then, we should observe no transfer to auditory number performance during the post-training testing. If we do see improvements in auditory number performance for the children who participated in visual ANS training, we can conclude that this task trains a non-visual ability that transfers to auditory number processing. For this reason, successful transfer to auditory number discrimination is a more powerful demonstration if we *do not* entirely control for confounding visual cues during training, as transfer can only occur if children attend to number.

**Figure 1:**
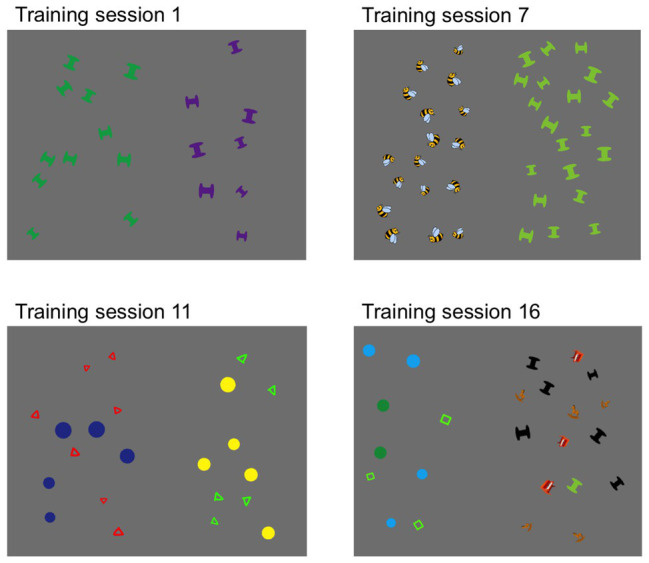
Sample stimuli from four representative visual approximate number system (ANS) training sessions, illustrating the increasing complexity of the stimuli. Children were asked to indicate whether the array on the left or the right had more items.

To support the possibility of ANS training causing a gradual improvement in performance, the ANS training was designed to become progressively harder, both within each training session and over the course of the 5 weeks. First, numerical discriminations became harder within each training block. Within each block, the comparison arrays were always presented in a pseudorandom fixed order of roughly increasing difficulty, i.e., children first saw arrays instantiating the easiest numerical ratio (3) and gradually progressed to arrays instantiating the hardest numerical ratio (1.11; for evidence that the order in which ratio discriminations are made affects ANS performance, see Odic et al., 2014; Wang et al., 2016, 2018). Second, to force children to make increasingly faster judgments, stimulus presentation duration decreased across the three blocks within each training session: 2,000 ms for the first block, 1,600 ms for the second block, and 1,200 ms for the third block. Importantly, all of these durations are too short for children to count the arrays exactly, requiring them to rely on their ANS throughout. Finally, stimulus complexity gradually increased across training sessions. Sessions began with simple shapes (circles and squares), then moved to more complex visual shapes (dumbbells and squiggles), and then to clipart images (toys and animals). Finally, children were initially presented with arrays that were homogeneous in item type (the left array contained items that were perceptually identical except for size, and the right array contained a different set of items that were perceptually identical except for size), but as children progressed through the training, they increasingly received trials containing heterogenous arrays (see Figure 1 and Supplementary Material). All of these factors were included in an attempt to scaffold children toward making faster and more accurate numerical discriminations, even when faced with complex scenes.

Prior to the start of ANS training, the experimenter introduced the task and instructed children to decide, as quickly as possible without counting, which array had more objects. Children were taught to use the “F” and “J” keys, marked with yellow and blue stickers on the laptop keyboard, to indicate which array contained more (left = “F”, right = “J”). Children received immediate feedback after every response: a high-pitched tone indicated a correct response and a low-pitched tone indicated an incorrect response. Children were told that, if they were unsure, they should make their best guess.

##### Phonological Awareness Training

Just as the ANS training involved three blocks within each training session, the phonological awareness training involved three blocks within each training session. Each of the three blocks presented a different mini-game from the Webber HearBuilder Phonological Awareness software (Super Duper® Publications): Rhyming, Syllable Blending, and Phoneme Blending. Stimuli were presented through the built-in speakers of the laptop. In the Rhyming block, children heard between 2 and 10 words spoken aloud (e.g., “mow,” “socks,” “toe,” and “wig”) paired with different images (e.g., a person mowing the lawn, socks, an image of a foot with an arrow pointing to the toe, and a wig) and had to quickly indicate which of the words rhymed by clicking on the corresponding pictures. In the Syllable Blending block, children heard a sequence of 2–5 syllables and had to identify which of 2–4 possible words would be formed if those syllables were blended in order, or they heard a spoken word and had to identify which of 2–4 sequences of unblended syllables corresponded to the word. In the Phoneme Blending block, children heard a sequence of 2–5 phonemes and had to identify which of 2–4 possible words would be formed if those phonemes were blended in order, or they heard a spoken word and had to identify which of 2–4 sequences of phonemes corresponded to the word. For all blocks, hovering over the stimulus icons replayed the corresponding auditory stimuli, and children could listen to them as often as they liked. For each correct answer, children heard music and received a star at the bottom of the screen. For each incorrect answer, they were told the correct answer and the next trial commenced. Children advanced to the next level of difficulty in the following training session if they answered at least 8 out of 10 questions correctly. Each game contained up to 22 levels of increasing task difficulty, and each level contained 10 trials. Task difficulty, in terms of the number of possible answer choices and length and familiarity of the target words, increased between levels and hence between training sessions. Completion of one level took about 5 min; hence, time spent on a block and total time spent on one training session were equated across the ANS and phonological awareness training conditions.

#### Pre‐ and Post-training Tasks

Both before and immediately after approximately 5 weeks of either visual ANS or PA training, each child completed a visual ANS precision task, an auditory ANS precision task, a standardized math assessment, and a standardized assessment of phonological awareness (mean delay between pre‐ and post-test: 37.78 days, *SD* = 8.19). All of these assessments were completed in children’s homes and were administered by one of two trained experimenters.

To ensure that the order in which the tasks were completed did not determine our results, we tested children in two different task orders. At both pre-training testing and post-training testing, half of the children in each training group first completed the standardized mathematics assessment, then the two ANS precision tasks, and then the standardized phonological awareness assessment. The other half first completed the phonological awareness assessment, followed by the two ANS precision tasks, and then the standardized mathematics assessment. The visual ANS precision task was always administered prior to the auditory ANS precision task (see below). It took children approximately 5–10 min to complete each ANS task, 20–30 min to complete the standardized mathematics assessment, and 20 min to complete the standardized phonological awareness assessment.

##### Visual ANS Precision Task

To measure the precision of children’s ANS for visual arrays, we administered a version of Panamath, a freely available non-symbolic numerical comparison task (www.panamath.org; Halberda et al., 2008; Libertus et al., 2011). Children sat at a table facing a laptop computer, with the experimenter at their side. The experimenter pointed to paper images of the cartoon character Grover and the character Big Bird, affixed to the left and right sides of the 13-in laptop screen. She told children that Grover had a box of blue balls and Big Bird had a box of yellow balls and that their job was to indicate who had more balls. The experimenter initiated each trial when children were attentive. On each trial, a collection of blue dots and a collection of yellow dots appeared simultaneously on the left and right sides of the screen, respectively. The dot arrays remained visible for 2,000 ms, after which a blank screen appeared and remained until children verbally indicated which character had more (e.g., saying “yellow”), at which point the experimenter pressed the corresponding key on an external keyboard (e.g., “y” for “yellow”). In pilot testing, we found that having the experimenter press the key following children’s verbal response produced a more accurate measure of the child’s response time (RT), because children sometimes had difficulty and became distracted as they tried to push the buttons themselves. The experimenter was seated to the side of the computer such that they could not see the stimuli; this ensured that the experimenter could not influence children’s response time or accuracy. Two sounds provided immediate response feedback throughout: a high-pitched tone indicated a correct response and a low-pitched tone indicated an incorrect response. Children were familiarized to these sounds on six practice trials during which the experimenter provided additional verbal feedback after any incorrect responses, to ensure that children understood the task and were motivated to participate. Following these practice trials, 42 test trials were presented.

The number of dots in each array (blue and yellow) ranged from 5 to 21. Six test trials were drawn from within each of seven numerical ratio bins: 1.11, 1.14, 1.17, 1.25, 1.5, 2, and 3, and were presented in randomized order. On half of the trials, the blue dots were more numerous, and on the other half, the yellow dots were more numerous. The dots in each array varied in size: their default radius was 60 pixels and the maximum between-dot variability in radius was ±35%. On half of all trials, the two arrays were equated for individual dot size (i.e., the average size of the dots in the blue array was equal to the average size of the dots in the yellow array), and on the other half, the two arrays were equated in cumulative surface area. These trial types were randomly intermixed throughout the testing session.

Children’s performance on the visual ANS precision task was measured in terms of accuracy (percent correct) and response time.

##### Auditory ANS Precision Task

To measure the precision of children’s ANS for auditory sequences, we administered an auditory non-symbolic number comparison task. Children again sat at a table in front of a 13-in laptop screen, with the experimenter at their side. Children and the experimenter wore headphones. Prior to the task, children first were told that Grover had a box of blue balls and that Big Bird had a box of yellow balls, and that their job was to indicate who had more balls. The experimenter then demonstrated that Grover’s balls were marked by sequences of low-pitched sounds – one for each ball – and that Big Bird’s balls were marked by sequences of high-pitched sounds – one for each ball. The experimenter initiated each trial when children appeared to be attentive. For each trial, the two sequences of sounds were presented in turn through the headphones. Children always heard Grover’s balls (i.e., the low-pitched sounds) presented on a consistent side and order (for example, always through the left side of the headphones, and always first). After the last sound in the second sequence, children gave a verbal response to the question “Who has more?” (e.g., “Big Bird”), after which the experimenter immediately pressed the corresponding key on an external keyboard. After each response, one of two different feedback pictures immediately appeared on the laptop screen: a smiley face indicated a correct response while a black rectangle indicated an incorrect response.

In order to scaffold children’s understanding of the task, we always administered the visual ANS precision task before the auditory ANS precision task because pilot testing showed that the auditory task was harder for children to understand than the visual task. Further, the first four practice trials of the auditory ANS precision task contained concurrent visual and auditory stimuli: a new yellow or blue dot appeared on the screen for each sound that was played, and all of the dots remained visible until the end of the practice trial. The experimenter also provided verbal feedback during these practice trials in order to ensure that children were motivated and understood the task. After the first four practice trials, the experimenter affixed a paper frame to the screen. This frame covered the entire screen except for a small rectangular area in the center where the feedback pictures appeared. In the next four practice trials and in the subsequent test trials, only the sounds were presented.

Following the eight practice trials, children completed 32 test trials. The number of sounds in each sequence (low-pitched and high-pitched) ranged from 5 to 16. Eight test trials were drawn from within each of four numerical ratio bins: 1.5, 2, 2.2, and 2.5 (a ratio of 1 would be equality). The ratios in the visual and auditory ANS tasks were chosen separately to ensure roughly equivalent total accuracy across both tasks, because previous work has shown that ANS tasks in which stimuli are presented sequentially are more difficult than ANS tasks in which the stimuli are presented simultaneously (Droit-Volet et al., 2008; Tokita and Ishiguchi, 2012; Tokita et al., 2013). On half of the trials, the high-pitched sounds were more numerous; on the other half, the low-pitched sounds were more numerous. The duration of the sounds in each sequence and the duration of the gaps between sounds varied within trial. The average sound and gap durations were normally distributed around each of the following values: 75 ms (range: 50–100 ms), 110 ms (70–150), 150 ms (90–190), 170 ms (110–220), and 190 ms (130–250). We used a standard deviation of 25 ms for all distributions, and distributions were truncated on the right or the left to avoid sequences that were too short or too long. On half of the trials, the two sequences were equated for average sound duration and inter-sound duration (i.e., the average sound duration and the gap durations in each sequence were equal), and on the other half of the trials, the two sequences were equated for total duration (i.e., the average sound and gap durations differed by the inverse of the numerical ratio difference). The two trial types were randomly intermixed throughout the testing session.

As in the visual ANS precision task, children’s performance on the auditory ANS precision task was measured in terms of accuracy (percent correct) and response time.

##### Standardized Math Assessment

To assess children’s mathematical abilities, we administered Form A of the Test of Early Mathematics Ability (TEMA-3; Ginsburg and Baroody, 2003) for our pre-training testing and Form B of the TEMA-3 for our post-training testing. The TEMA-3 is normed for children between the ages of 3 years 0 months and 8 years 11 months and is comprised of 72 items testing children’s counting (e.g., “Count with me. 1, 2, 3, 4, and then comes?”), calculation skills (e.g., “Joey has 1 token, and he gets 2 more. How many does he have altogether?”), numeral literacy (e.g., “What number is this?” while pointing to a printed Arabic numeral), and understanding of number concepts such as the cardinality principle (e.g., “I’m going to count some tokens. Next, I’m going to move the tokens around. Then, without counting, you tell me how many tokens there are”). As specified in the Experimenter’s Manual, testing started at a specific item based on a child’s age and continued until the child answered five consecutive items incorrectly (ceiling). Items before the start item were administered in backward order if a child had not responded correctly to five consecutive items (basal) when the ceiling was reached. All items before the basal were counted as correct even though they were not administered. Math abilities were measured as raw scores on the TEMA-3. Note that we used raw scores rather than standardized scores because standardized TEMA scores are less sensitive to changes over short periods of time since they are age-normed over 3-month intervals. This standardization would cause some but not all of the children in our sample to shift from one age bracket to the next between pre‐ and post-training testing, creating uneven shifts in standard scores.

##### Standardized Phonological Awareness Assessment

To assess children’s phonological awareness, we administered the Comprehensive Test of Phonological Processing (CTOPP; Wagner et al., 1999). Because most of our participants were between 5 and 6 years old, we administered the eight subtests of the CTOPP appropriate for this age range. These tests include Elision (i.e., the ability to remove phonological segments from spoken words to form other words), Rapid Color Naming (i.e., the ability to rapidly name the colors of squares presented on a page), Blending Words (i.e., the ability to synthesize sounds to form words), First and Last Sound (i.e., the ability to identify the first and last sound of a spoken word), Rapid Object Naming (i.e., the ability to rapidly name common objects presented on a page), Memory for Digits (i.e., the ability to repeat spoken numbers in the correct order), and Non-word Repetition (i.e., the ability to repeat spoken non-words accurately). Children took about 20 min to complete the CTOPP. Because some of our participants were older or younger than the age range for which our CTOPP subtests were standardized, we calculated a CTOPP composite score by calculating *z*-scores for each subtest individually and then averaging *z*-scores across all subtests.

#### General Cognitive Abilities

##### IQ

We measured children’s IQ about 10 months prior to the pre-training testing using the Primary Test of Nonverbal Intelligence (PTONI; Ehrler and McGhee, 2008). The PTONI has been normed for children between 3 years 0 months and 9 years and 11 months and takes 5–15 min to complete. The PTONI asks children to look at a series of pictures on each page in a picture book and point to the one picture that does not belong with the others. Items are arranged in order of difficulty, whereby early items measure lower order reasoning (e.g., visual and spatial perception) and later items measure higher order reasoning abilities (e.g., analogical thinking, sequential reasoning, and category formulation). Children’s performance was measured using age-normed standard scores.

##### Inhibitory Control

We measured children’s inhibitory control abilities about 16 months prior to the pre-training testing using the Conners’ Kiddie Continuous Performance Test Version 5 (K-CPT; Conners, 2006). The K-CPT has been normed for children ages 4 and 5 years and takes 7 min to administer. Children see a stream of images on a computer screen and are asked to press a button every time they see an image of anything other than a soccer ball; hence, children must inhibit a pre-potent response on the critical trials in which a ball is shown. Because some of our participants were outside of the age range for which the K-CPT has been normed, we report the percentage of commission errors (i.e., responses to the non-target soccer ball) as a measure of inhibitory control.

## Results

Improvements in ANS precision from pre‐ to post-training may be seen in either faster RT or more accurate responses (percent correct), or both. Planned comparisons revealed that children in the ANS training group were significantly faster in the visual ANS precision task post-training compared to pre-training, *t*(32) = 7.71, *p* < 0.001, but they were less accurate, *t*(32) = 2.69, *p* = 0.01. Children in the PA training group were faster in the visual ANS precision task post-training than pre-training, *t*(34) = 5.38, *p* < 0.001, and their accuracy remained unchanged, *t*(34) = −0.16, *p* = 0.88. To take these speed/accuracy tradeoffs into account, we calculated an efficiency measure based on RT and accuracy (efficiency = RT/accuracy; Townsend and Ashby, 1978, 1983), whereby a larger efficiency value indexed worse ANS performance. To simplify our analyses, we used this measure of ANS efficiency in all further analyses (for discussion of the importance of considering reaction time and accuracy when measuring numerical approximation ability, see Park and Starns, 2015).

### The Relation Between Visual and Auditory ANS Efficiency Before Training

**Figure 2:**
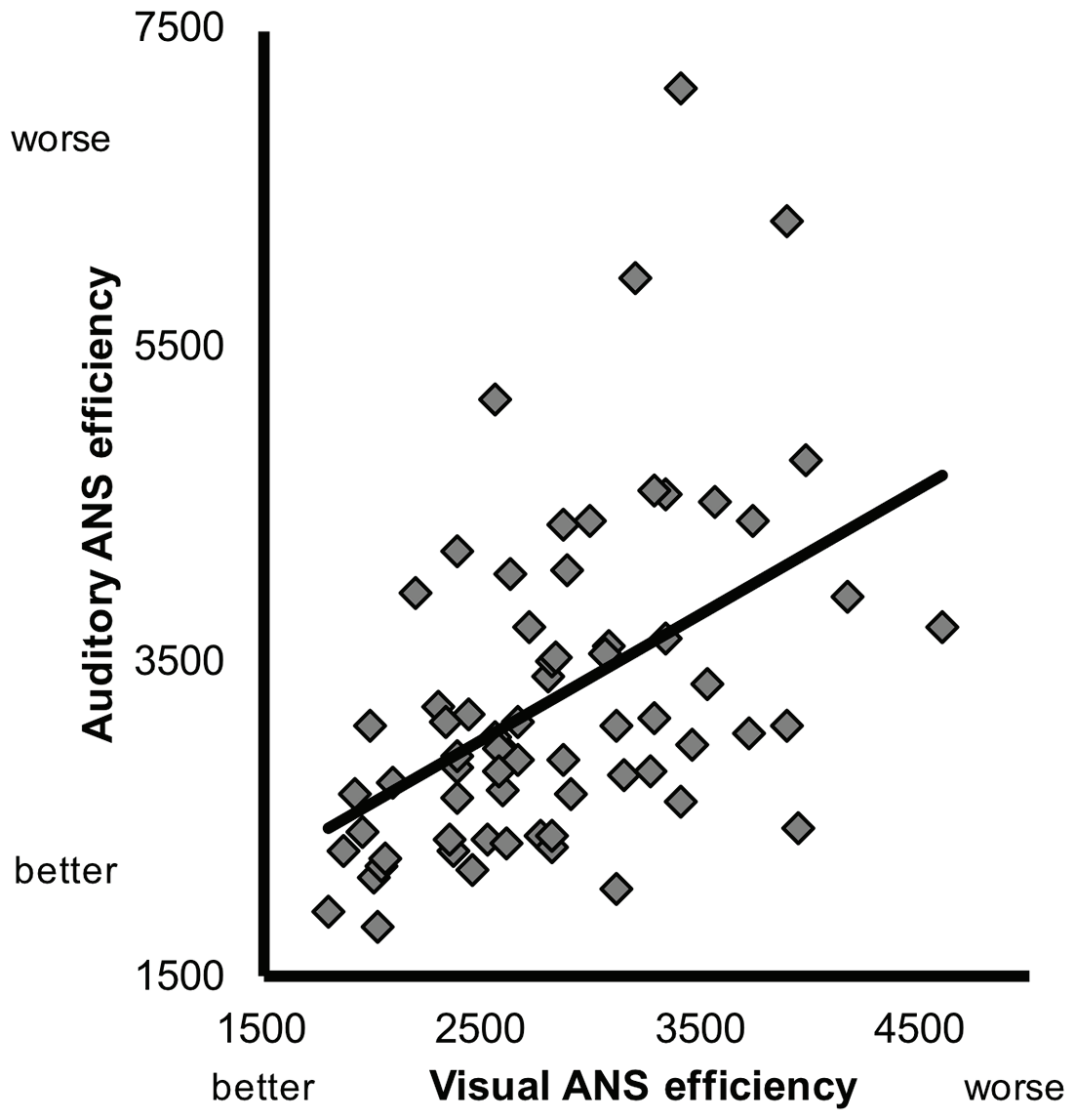
Scatterplot depicting the relation between visual and auditory ANS efficiency prior to training.

First we investigated the relation between individual differences in visual and auditory ANS efficiency. As discussed in the introduction, because visual cues and auditory cues to number occur in different sensory modalities, responding to visual cues alone (e.g., responding to convex hull without ever creating an amodal number representation) should not readily transfer across modalities or lead to improved auditory number precision. An indication that humans do have amodal number representations would come from a correlation of individual differences in visual and auditory ANS efficiency. Indeed, we found a significant zero-order correlation between visual and auditory ANS efficiency prior to training (*r* = 0.48, *p* < 0.001; Figure 2).

To explore whether this relation held even when controlling for other factors, we ran a hierarchical linear regression. In the first step of the regression model, we entered age at the time of testing, IQ, and inhibitory control as potential predictors of auditory ANS efficiency. In the second step, we added visual ANS efficiency to assess whether this captured additional variance in auditory ANS efficiency beyond age, IQ, and inhibitory control. Data from one child had to be excluded because the residual was more than 2.5 standard deviations above the mean. As can be seen in Table 1, visual ANS efficiency explained a significant amount of variance in auditory ANS efficiency above and beyond age, IQ, and inhibitory control, *R* ^2^_change_ = 0.13, *F*_change_(1,54) = 9.32, *p* < 0.01. This supports the existence of an amodal ANS and suggests that individual differences in ANS precision are not solely due to individual differences in sensitivity to visual dimensions, like density, area, and perimeter or, in the case of auditory stimuli, the duration and rate of presentation. Finally, the shared need for inhibitory control cannot explain the observed relation between visual and auditory ANS precision, because our analyses showed significant relations between visual and auditory ANS efficiency even after controlling for inhibitory control (as well as age and IQ).

**Table 1:** Summary of hierarchical linear regression analysis for variables predicting auditory ANS efficiency prior to training.

	Step 1			Step 2		
Variable	*B*	*SE B*	*β*	*B*	*SE B*	*β*
Age	−1.24	0.49	−0.32^*^	−0.82	0.48	−0.21
IQ	7.02	6.25	0.14	6.59	5.83	0.14
Inhibitory control	0.79	4.39	0.02	0.06	4.10	0.002
Visual ANS efficiency				0.56	0.18	0.37^**^
*R* ^2^		0.13			0.26	
*F* for change in *R* ^2^		2.72			9.32^**^	

* *p* < 0.05;

** *p* < 0.01.

### Is the ANS Related to Formal Math? Relation Between ANS Efficiency and Math Ability Prior to Training

**Figure 3:**
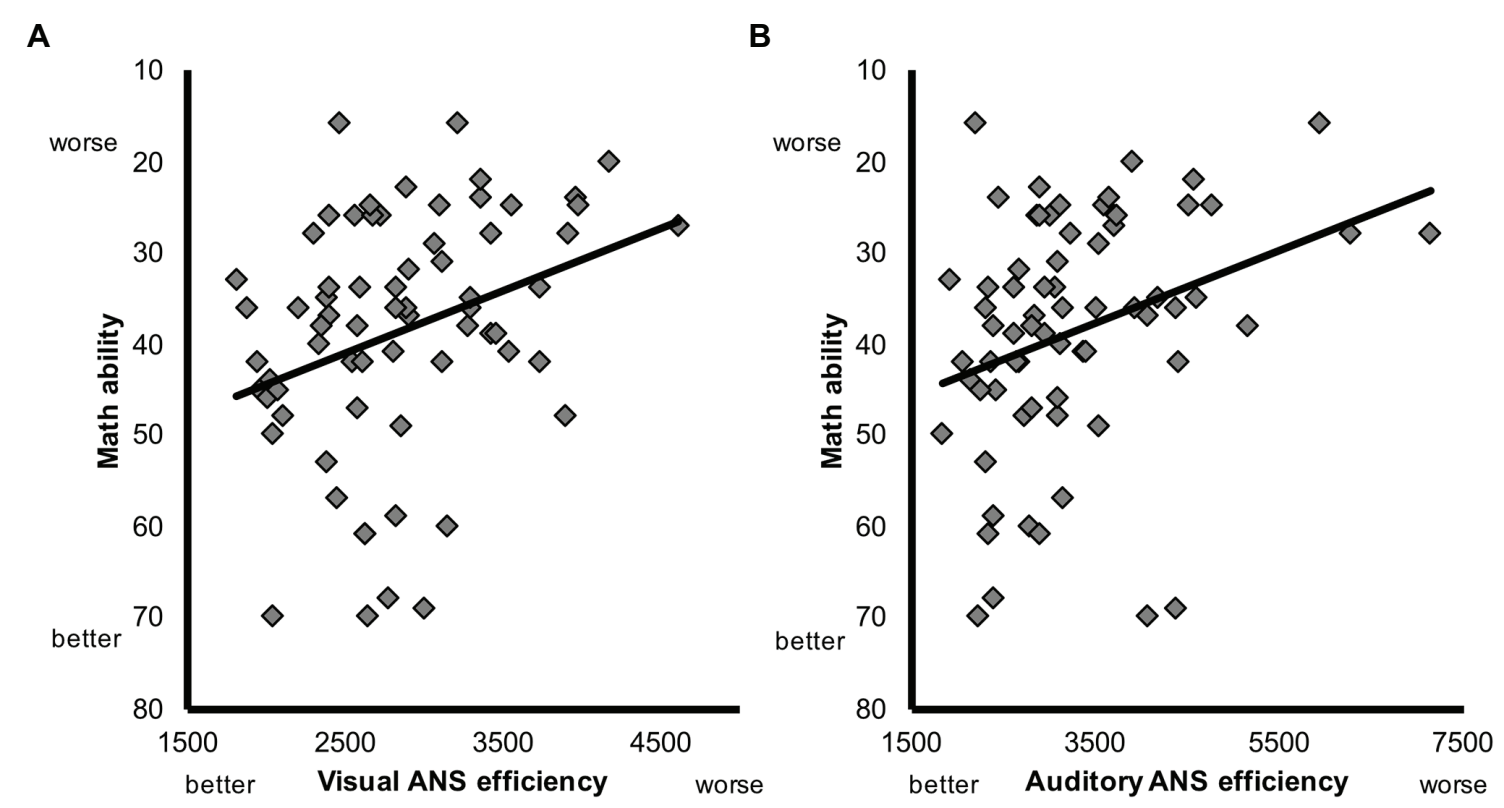
Scatterplot depicting the relation between math ability [Test of Early Mathematics Ability (TEMA) raw score], **(A)** visual ANS efficiency, and **(B)** auditory ANS efficiency prior to training.

To assess the relation between children’s ANS efficiency and math ability prior to training, we first calculated the zero-order correlations between TEMA scores and visual and auditory ANS efficiency. We found significant correlations between math and visual ANS efficiency (*r* = −0.32, *p* < 0.01), and math and auditory ANS efficiency (*r* = −0.31, *p* < 0.01). Higher visual and auditory ANS efficiency each were associated with greater math ability (Figure 3).

Next, to test whether ANS efficiency regardless of modality predicted math ability above and beyond age, IQ, and inhibitory control, we conducted a hierarchical linear regression analysis. In the first step, we entered age at the time of testing, IQ, and inhibitory control as potential predictors and TEMA scores as the outcome. In the next step, we added the visual and auditory ANS efficiency measures to ask whether they predicted additional variance in TEMA scores above and beyond age, IQ, and inhibitory control. Importantly, since visual and auditory ANS efficiency are significantly correlated, we expected them to jointly predict additional variance in TEMA scores, but not necessarily to be unique predictors. Data from two children had to be excluded from these analyses because their standard residuals were more than 2.5. As expected, ANS efficiency prior to training explained a significant amount of variance in math ability above and beyond age, IQ, and inhibitory control, *R* ^2^_change_ = 0.07, *F*_change_(2,52) = 3.87, *p* < 0.03, i.e., an additional 7% of the variance in children’s math ability was explained by variation in visual and auditory ANS efficiency. As can be seen in Table 2, neither visual nor auditory ANS efficiency was a unique predictor of math ability above and beyond the other ANS efficiency measures – this is to be expected because most of the variance in math scores accounted for by visual ANS efficiency was also accounted for by auditory ANS efficiency. Thus, this is exactly the pattern one would predict if performances in the visual and auditory ANS precision tasks both index the precision of an amodal representational system.

**Table 2:** Summary of hierarchical linear regression analysis for age, IQ, inhibitory control, and visual and auditory ANS efficiency predicting math ability prior to training.

	Step 1			Step 2		
Variable	*B*	*SE B*	*β*	*B*	*SE B*	*β*
Age	0.03	0.005	0.67^***^	0.03	0.005	0.56^***^
IQ	0.04	0.07	0.07	0.06	0.06	0.09
Inhibitory control	−0.04	0.05	−0.10	−0.04	0.05	−0.08
Visual ANS efficiency				−0.003	0.002	−0.14
Auditory ANS efficiency				−0.002	0.001	−0.21
*R* ^2^		0.45			0.52	
*F* for change in *R* ^2^		14.62^***^			3.87^*^	

* *p* < 0.05;

*** *p* < 0.001.

### The Effects of Training on Visual and Auditory ANS

Our next set of questions concerned the effects of training. Before comparing the effects of the two types of training on children’s visual and auditory ANS precision, math performance, and phonological awareness, we wanted to make sure the groups did not differ on these measures before the training began. We, therefore, compared the groups’ pre-training performance on the visual and auditory ANS tasks, TEMA (math) raw scores, and CTOPP (phonological awareness) composite scores (see Table 3). We found no significant differences between the two groups on any of these measures (all *p*s > 0.25), except for a marginally significant difference in visual ANS RT (*p* = 0.052), which was due to faster RTs in the ANS training group compared to the PA training group. Note that this trend would make it harder for us to observe the improvements in RT that we predict for the ANS training group. In addition, children in the two training groups did not differ in age at the time of pre-training testing, *t*(66) = −0.88, *p* = 0.38, or post-training testing, *t*(66) = −0.90, *p* = 0.37. Finally, children in the two training groups did not differ in general intelligence (ANS training group: *M* = 118.69, *SD* = 18.18; PA training group: *M* = 126.91, *SD* = 19.71), *t*(64) = 1.76, *p* = 0.08, or inhibitory control (ANS training group: *M* = 48.16, *SD* = 26.73; PA training group: *M* = 55.40, *SD* = 26.10), *t*(60) = 1.08, *p* = 0.29.

**Table 3:** Pre-training and post-training scores for the visual and auditory ANS precision tasks, math ability (raw scores on the TEMA-3), and phonological awareness (composite *z*-score on the CTOPP), presented separately for the two training groups.

	ANS training group means (SD)		PA training group means (SD)	
	Pre-training	Post-training	Pre-training	Post-training
Age in days	2244.79 (253.18)	2282.00 (252.90)	2192.63 (234.52)	2228.91 (235.48)
Visual ANS accuracy	86.31% (7.21)	83.07% (6.05)	85.55% (6.91)	85.73% (5.46)
Visual ANS RT	2306.22 (416.45)	1801.64 (299.52)	2534.64 (524.58)	2129.65 (337.42)
Visual ANS efficiency	2692.49 (547.30)	2188.33 (442.87)	2977.00 (652.75)	2499.69 (466.50)
Auditory ANS accuracy	85.89% (13.04)	85.32% (10.69)	85.88% (9.26)	82.45% (9.09)
Auditory ANS RT	2620.50 (675.72)	2488.77 (550.16)	2827.30 (494.04)	2914.51 (793.25)
Auditory ANS efficiency	3185.25 (1236.01)	3001.36 (925.41)	3352.41 (811.29)	3590.86 (1096.91)
TEMA-3	40.39 (14.22)	42.61 (14.13)	36.74 (11.81)	38.37 (11.78)
CTOPP	0.02(0.55)	0.05(0.50)	−0.02(0.40)	0.12(0.39)

**Figure 4:**
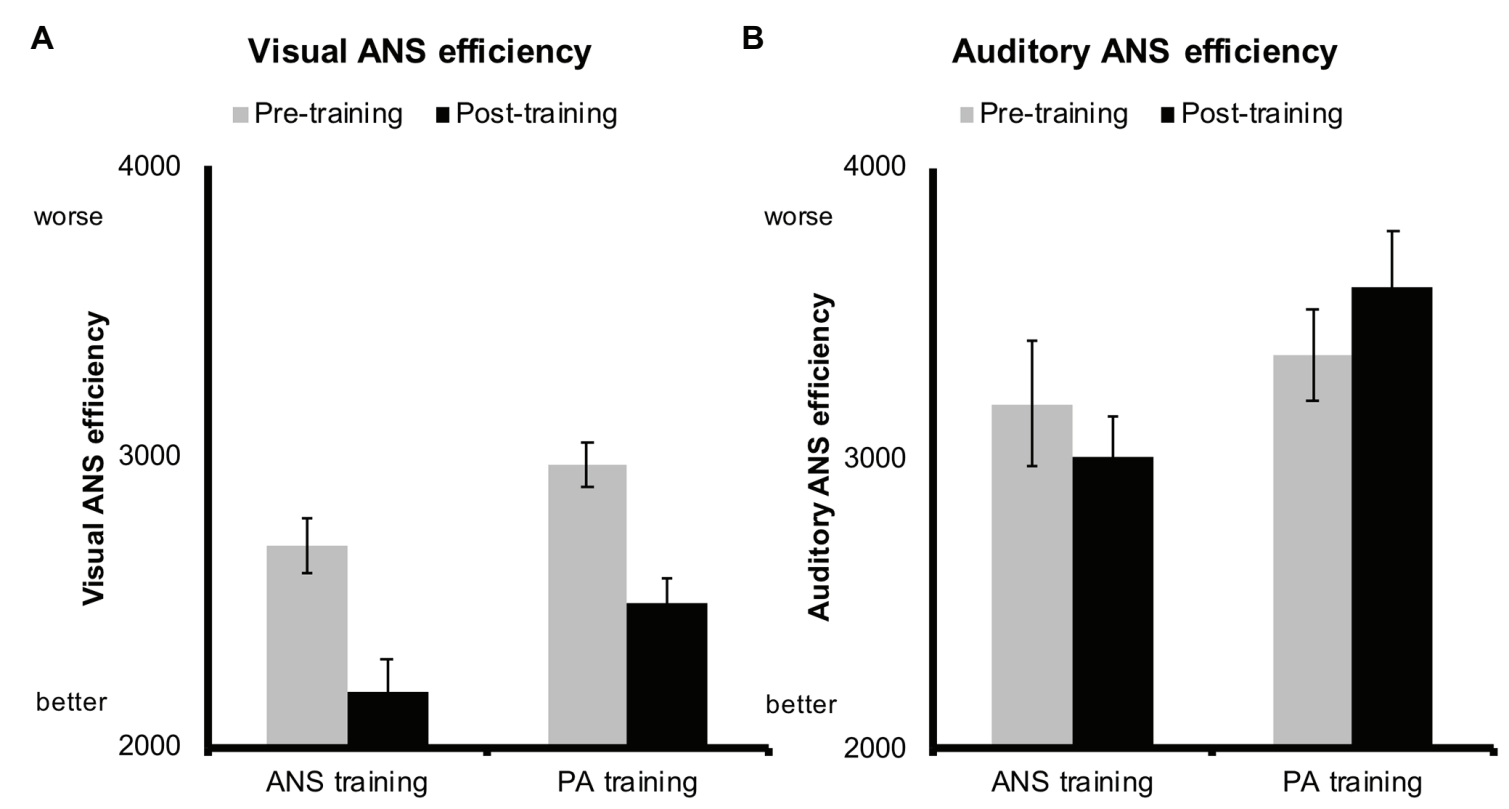
**(A)** Visual and **(B)** auditory ANS efficiency before training (gray bars) and after training (black bars) for the ANS training group and the phonological awareness (PA) training group. Error bars depict standard errors. Efficiency is calculated as response time (RT)/accuracy, i.e., a larger value reflects worse ANS performance.

To assess the impact of the two types of training on visual and auditory ANS efficiency, we conducted a repeated-measures ANOVA for each modality with training group (ANS and PA) and testing point (pre-training and post-training) as factors. We included age at post-training testing, IQ, and inhibitory control as covariates to control for the effects of these variables. For visual ANS efficiency, we found a significant main effect of training group, *F*(1,55) = 7.02, *p* = 0.01, that was due to significantly greater efficiency in the ANS training group compared to the PA training group (Figure 4). No other main effects or interactions reached significance when controlling for children’s age, IQ, and inhibitory control (all *F*s < 0.73, *p*s > 0.39).

For auditory ANS efficiency (i.e., our crucial transfer task), we found a significant interaction between training group and testing point, *F*(1,55) = 4.60, *p* = 0.04. As seen in Figure 4, children in the ANS training group – who had received 5 weeks of experience in a visual ANS task – tended to improve in auditory ANS efficiency from pre-training to post-training, while children in the PA training group tended to drop in auditory ANS efficiency. This interaction suggests that visual ANS training may transfer to auditory ANS efficiency, while auditory phonological awareness training, in our sample, was associated with a decline in auditory ANS efficiency. However, neither group showed a significant change in auditory ANS efficiency from pre‐ to post-training (ANS training: *t*(32) = 1.23, *p* = 0.23; PA training: *t*(33) = −1.36, *p* = 0.18).

### Does ANS Training Transfer to Formal Math? Effects of Training on Math Ability

**Figure 5:**
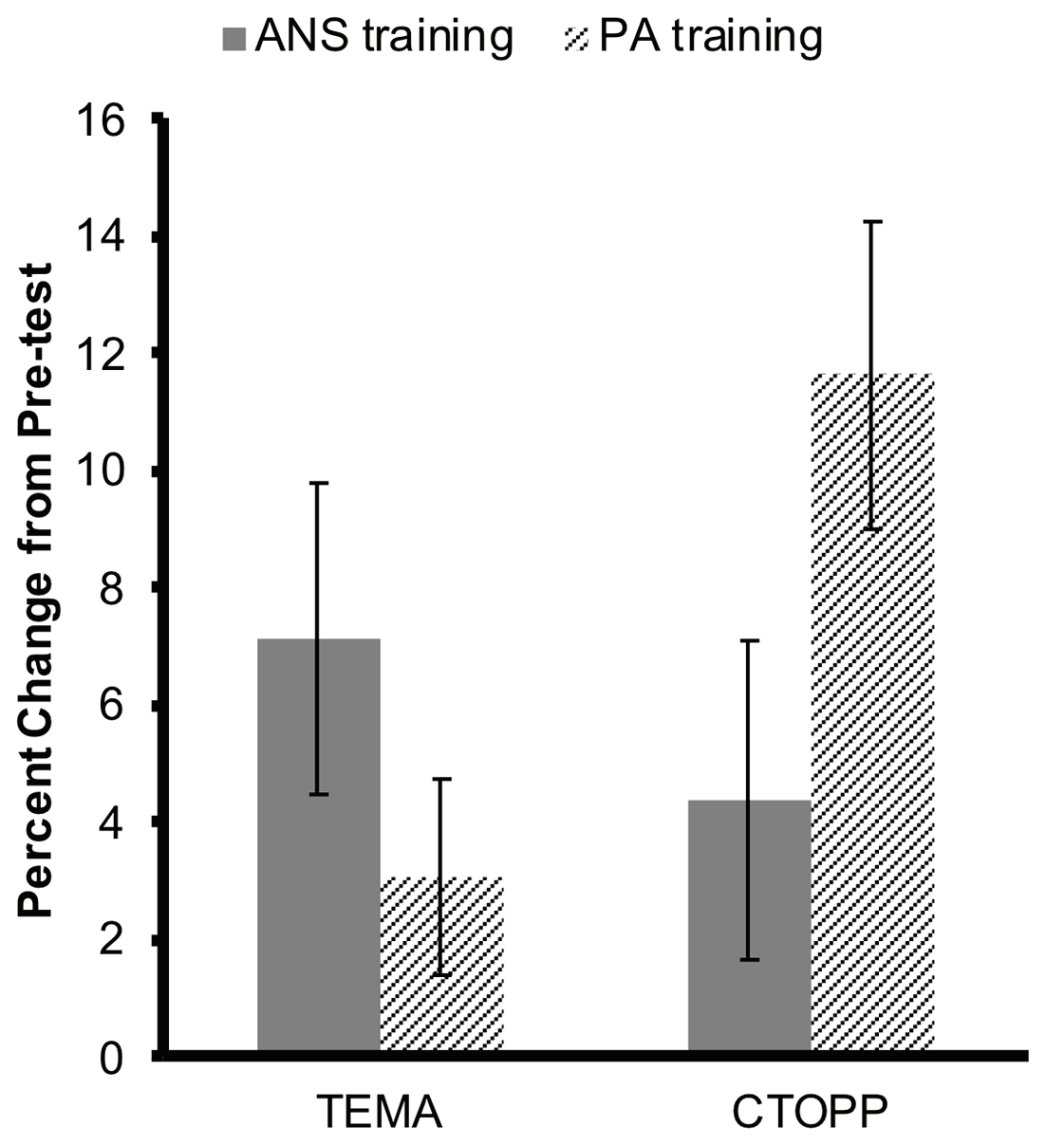
Percent change from pre-training performance on the TEMA and the Comprehensive Test of Phonological Processing (CTOPP) for children in the ANS and PA training groups, respectively. Error bars depict standard errors of the mean.

To ask whether training in either visual ANS discrimination or in phonological awareness improved either math performance or phonological awareness (while controlling for pre-training performance), we calculated the percent change in children’s performance from pre-training to post-training, relative to pre-training performance [i.e., (post-training − pre-training)/(pre-training × 100)] for children’s raw TEMA scores and CTOPP composite scores. Data from one child had to be excluded because the percent change in TEMA was more than 2.5 standard deviations above the mean. A repeated-measures ANOVA examining the effects of training Group (ANS and PA) and task (TEMA and CTOPP) on these percent change scores revealed a significant interaction between training group and task, *F*(1,65) = 6.00, *p* = 0.02 (Figure 5). This interaction remained significant even when age at post-training testing was included as a covariate, *F*(1,64) = 5.49, *p* = 0.02. However, when adding IQ and inhibitory control as additional covariates, the interaction was no longer significant, *F*(1,54) = 1.64, *p* = 0.21.

One-sample *t*-tests comparing percent change from pre-training to post-training revealed that children in the ANS training group showed significant improvements in math, *t*(32) = 2.68, *p* = 0.01, but not phonological awareness, *t*(32) = 1.60, *p* = 0.12, whereas children in the PA training group showed significant improvements in phonological awareness, *t*(33) = 4.22, *p* < 0.001, but not math, *t*(33) = 1.75, *p* = 0.09. This suggests that visual ANS training is associated with improvements in symbolic math performance but not phonological awareness, while phonological awareness training is associated with improvements on a standardized phonological awareness assessment but not math. However, there was no significant difference in changes in math between the ANS and the PA training groups, *t*(65) = −1.29, *p* = 0.20, whereas the difference in changes in phonological awareness between the groups was marginally significant, *t*(65) = 1.87, *p* = 0.07. Finally, in the ANS training group, the change in math was not significantly different from the change in phonological awareness, *t*(32) = −0.84, *p* = 0.41, while this difference was significant in the PA training group, *t*(33) = 2.63, *p* = 0.01.

## Discussion

Prior work on the ANS has suggested that it may represent number in an amodal fashion, readily taking both sounds and visual objects as input, and that these intuitive number representations may be linked to school math performance. On the other hand, it has also been proposed that visual ANS tasks simply measure sensitivity to visual area, visual density, or some other non-numeric visual feature, and that the ANS, therefore, has no inherent connection to school math ability other than being linked by general visuospatial performance. To address this, we investigated the effect of visual ANS training on subsequent auditory ANS performance and on subsequent standardized math performance. We report four main findings: (1) individual differences in children’s visual ANS performance correlated with individual differences in auditory ANS performance, even when controlling for age, IQ, and inhibitory control; (2) ANS precision measured in both the auditory and visual modality predicted math ability prior to training, even when controlling for age, IQ, and inhibitory control; (3) 5 weeks of training on a visual ANS task was associated with greater improvements in auditory ANS precision than 5 weeks of training on phonological awareness tasks; and (4) 5 weeks of training on a visual ANS task was associated with significant improvements in subsequent math performance but not phonological awareness, whereas 5 weeks of training on a phonological awareness task was associated with significant improvements in subsequent phonological awareness but not math performance.

These findings extend previous studies by demonstrating that not only can individuals represent visual and auditory number, and integrate numerical representations of visual and auditory arrays (Barth et al., 2005, 2006, 2008), but, in addition, that children with sharper numerical precision in one sensory modality also have sharper numerical precision in another modality. These findings are in line with recent results by Anobile et al. (2018), who found that 7‐ to 11-year-old children’s performance on a visual non-symbolic number estimation task significantly correlated with their ability to estimate numbers of tones even when controlling for children’s age and nonverbal IQ. Here, we show that the relation between visual and auditory number comparison abilities cannot be explained by a shared need to inhibit irrelevant perceptual information, such as the cumulative surface area of visual arrays or the total duration of auditory sequences, because visual ANS precision predicted auditory ANS precision even when controlling for inhibitory abilities (as well as age and IQ). This suggests that ANS tasks measure an abstract numerical sense, as there is no reason to expect individual differences in the ability to represent visual aspects of a scene to correlate with individual differences in the ability to represent temporal aspects of an auditory sequence.

In addition, our findings add to the growing body of work demonstrating that ANS representations are malleable and can be improved through training. Previous research found that training in a visual ANS task improved ANS performance in adults (DeWind and Brannon, 2012) and children (Odic et al., 2014; Wang et al., 2016, 2018). Our present results suggest that these improvements may transfer from one sensory modality to another – such that practice in a visual numerical approximation task leads to better performance in an auditory numerical approximation task, at least relative to practice in phonological awareness. The training effect we observed here was not strong (i.e., children’s change in pre‐ vs. post-training performance was small), hence the training we employed here is not a candidate for practical interventions intending to improve children’s numerical competence. Rather, the contribution of our training results consists in providing evidence as to the nature of the ANS – if experience with visual approximation even weakly affects auditory approximation, this implicates the existence of an abstract sense of numerosity.

It is important to note that our results do not speak to the mechanisms by which observers extract numerical information from visual and auditory displays. Even though many studies have considered possible mechanisms that might support the extraction of numerical information from visual displays (Ginsburg and Nicholls, 1988; Vos et al., 1988; Allik and Tuulmets, 1991; Dehaene and Changeux, 1993; Durgin, 1995; Verguts and Fias, 2004; Sophian and Chu, 2008; Tokita and Ishiguchi, 2010a; Dakin et al., 2011; Gebuis and Gevers, 2011; Gebuis and Reynvoet, 2012; Tibber et al., 2013; Odic and Halberda, 2015; Odic, 2018), the exact algorithms used by observers have not yet been uncovered. In the visual domain, key input to the ANS may very well involve area, density, convex hull, ratios of activity in various spatial frequency channels, or other visual characteristics that correlate with number. Representing the approximate number of items in an array can only happen as a result of the physical effects that the array has on the observer. So, in this sense, it is trivially true that the ANS computes over some combination of visual features in visual tasks, and it remains an important and interesting challenge to determine which dimensions these algorithms compute over and in which contexts (Halberda, 2019). Similar questions will no doubt arise for determining the algorithms that support the extraction of approximate number from auditory sequences. But the correlations we find across modalities highlight the shared numerical nature of the ANS representations that are derived from these modality-specific features.

Our findings that visual and auditory ANS precision predict math ability even when controlling for age, IQ, and inhibitory control suggest that the link between ANS performance and math abilities is not based solely on visuospatial processes (Soltesz et al., 2010; Gilmore et al., 2011; Gebuis and Reynvoet, 2012; Sasanguie et al., 2013) or inhibitory control (Fuhs and McNeil, 2013; Gilmore et al., 2013). Rather, the ANS appears to be amodal (i.e., it can generate numerical estimates of either simultaneously presented visual stimuli or serially presented auditory stimuli), and the precision of these modality-independent approximate number representations relates to school math abilities. Our findings might seem to contradict recent findings by Anobile et al. (2018), who reported that performance on an approximate number estimation task using simultaneously presented visual stimuli, but not sequentially presented visual or auditory stimuli, correlated with children’s math abilities. However, these differences may be due to the fact that Anobile and colleagues asked their participants to make explicit numerical estimates of collections of dots or sequences of sounds (i.e., to map representations of approximate numerosities to exact number words), rather than simply compare them as in our case. It remains an open question which task places the greater task demands on the subject and which allows for a greater influence of executive functions, response strategies, and bias. Finally, it is possible that our task order, i.e., the fact that the auditory ANS task always followed the visual ANS task, may have led children to use other strategies to complete the auditory ANS task than in the study by Anobile and colleagues and that these variations in strategy use may explain the different associations with children’s math abilities.

Finally, our finding that visual ANS training is associated with improvements in math performance but not phonological awareness adds to the growing literature on the causal link between the ANS and math ability. Recently, Park and Brannon (2013) showed that, after 2 weeks of training on a non-symbolic addition and subtraction task (in which observers mentally added or subtracted two dot arrays), adults improved significantly on a symbolic arithmetic test. In contrast, adults did not improve in symbolic math if their training involved simple comparison (choosing the greater of two dot arrays; Park and Brannon, 2014). Young children also show effects of ANS training. First, preschool-aged children improve in some aspects of math performance following experience mentally adding and subtracting approximate quantities (Park et al., 2016). Hyde et al. (2014) found that brief training in non-symbolic number addition, but also in non-symbolic number comparison, improved children’s subsequent arithmetic performance within the same testing session. And, Wang et al. (2016) showed immediate transfer from a scaffolded ANS comparison training to a standardized math task, but not a verbal task.

Aside from the ANS supporting math abilities, there is also evidence to suggest that experience with symbolic number leads to improvements in the ANS. For example, preschool-aged children’s understanding of cardinality and symbolic number as well as their general math abilities predict later ANS acuity (Mussolin et al., 2014; Elliott et al., 2019), and 5-year-old children’s symbolic number comparison abilities were a significant predictor of growth in non-symbolic number comparison skills between the beginning and end of the school year (Lyons et al., 2018). In addition, adults with more mathematical education also perform better on nonverbal numerical approximation tasks (Piazza et al., 2013; Lindskog et al., 2014). Although it will require future work to characterize the scope and duration of the bidirectional relation between the ANS and math ability, the accumulating body of evidence suggests that there is a causal relation between intuitive approximate number representations and the symbolic math abilities that children typically begin to acquire during the process of formal schooling.

While our results provide evidence for amodal approximate number representations and their association with children’s math abilities, we acknowledge a number of limitations in our current study. First, it is unclear whether or to what extent our evidence will be important for informing the design of interventions aimed at improving math; for example, our study was not designed as a randomized controlled trial. Rather, the broad aim here was to further our understanding of the relations between core systems of thought and culturally constructed abilities. Second, our auditory ANS task always followed the visual ANS task and used simultaneous visual and auditory stimuli during the practice trials to ensure that children understood the task. One possible concern is that children could solve the auditory-only trials by imagining a dot appearing as they heard each tone – making this, in effect, an imaginary visual ANS task. However, evidence suggests that children presented with difficult approximate numerical discriminations perform better when presented with redundant visual and auditory input, compared to only visual input, suggesting that auditory tones are not simply treated as indicating the presence of visual objects (Posid and Cordes, 2019). Furthermore, people blind from birth succeed at purely auditory ANS tasks (Kanjlia et al., 2018), showing that ANS representations can be generated in the total absence of any visual experience. Still, despite the above findings, it is impossible to entirely rule out an account by which visualization plays some role in the processing of auditory input – something that is currently the topic of much ongoing debate (De Volder et al., 2001; Amedi et al., 2005; Vetter et al., 2014). Thus, while it is unlikely, we cannot rule out that children were constructing mental images of the tone sequences in the test trials and performing comparisons on these mental images; it is unclear whether visualization of purely imagined stimuli is related to the kinds of visuospatial abilities discussed as contributing to mathematical competence. Finally, while not uncommon, the fact that our IQ and inhibitory control measures were administered at least 10 months prior to training may have reduced the association between these measures, ANS precision and math abilities. In addition, IQ and inhibitory control were only measured once leaving open the possibility that they may have improved over the course of the training duration and possibly to different degrees depending on training condition. Future work should assess general cognitive abilities in closer temporal proximity and prior to as well as after training to test whether the link between an amodal ANS and math and training-related effects remain significant even when controlling for general cognitive abilities.

In sum, in the present study, we found that children’s ANS precision correlates across the visual and auditory modalities and that visual ANS training was associated with greater improvements in auditory ANS precision compared to auditory phonological awareness training. Finally, visual ANS training was also associated with significant improvements in symbolic math ability but not phonological awareness. These results support the existence of a modality-independent ANS and suggest a causal link from the ANS to math ability that cannot be explained by age, intelligence, or inhibitory control.

## Data Availability Statement

The datasets generated for this study are available on request to the corresponding author.

## Ethics Statement

The studies involving human participants were reviewed and approved by Johns Hopkins University Institutional Review Board. Written informed consent to participate in this study was provided by the participants’ legal guardian/next of kin.

## Author Contributions

ML, LF, and JH designed the study. ML collected the data. ML, DO, LF, and JH analyzed the data and wrote the paper. All authors contributed to the article and approved the submitted version.

### Conflict of Interest

The authors declare that the research was conducted in the absence of any commercial or financial relationships that could be construed as a potential conflict of interest.
