# Supplementary material for: Effects of Visual Training of Approximate Number Sense on Auditory Number Sense and School Math Ability
Source: Front Psychol. 2020 Aug 27;11:2085. doi: 10.3389/fpsyg.2020.02085 (PMC7481447; doi:10.3389/fpsyg.2020.02085)
Supplement: Supplementary file 1 [file Data_Sheet_1.docx]

**Supplemental Methods**

**Approximate Number System (ANS) Training**

To increase task difficulty across training sessions, we varied stimulus categories from simple (circle, square) to more complex (dumbbell, squiggle) colored shapes to clipart images (toys, animals) as well as the homogeneity of the objects in each array and between arrays. In all three blocks of the initial two sessions, children compared homogeneous arrays from the same stimulus category (e.g., one array contained multiple images of identical clipart toy cars and the other array contained multiple images of identical clipart teddy bears). In the following five sessions, for one or two out of the three blocks children compared homogeneous arrays from different stimulus categories (e.g., one array contained green squares and the other array contained clipart toy airplanes). The remaining 1-2 blocks were composed of identical stimulus categories similar to the first two sessions. In the next three sessions, one of the three blocks contained homogeneous arrays from different stimulus categories as described before and the other two blocks contained heterogeneous arrays (e.g., circles and squares) but both arrays contained the same categories distinguished by different colors and spatial separation. In the final six sessions, one of the three blocks contained heterogeneous arrays but both contained the same categories as described before and the other two blocks contained heterogeneity both within each array as well as between arrays (e.g., one array contained circles of multiple different colors and clipart animals of different kinds, and the other array contained squares of multiple different colors and clipart toys of different kinds). All arrays were clearly separated on the left and the right side of the screen and always contained different colored images even when stimuli were homogeneous in category.
